# CHIR99021 Augmented the Function of Late Endothelial Progenitor Cells by Preventing Replicative Senescence

**DOI:** 10.3390/ijms22094796

**Published:** 2021-04-30

**Authors:** Vinoth Kumar Rethineswaran, Da Yeon Kim, Yeon-Ju Kim, WoongBi Jang, Seung Taek Ji, Le Thi Hong Van, Ly Thanh Truong Giang, Jong Seong Ha, Jisoo Yun, Jinsup Jung, Sang-Mo Kwon

**Affiliations:** 1 Convergence Stem Cell Research Center, Pusan National University, Yangsan 50612, Korea; vinrebha@gmail.com (V.K.R.); ekdus0258@gmail.com (D.Y.K.); twou1234@nate.com (Y.-J.K.); jangwoogbi@naver.com (W.J.); jst5396@hanmail.net (S.T.J.); lethihongvan25121978@gmail.com (L.T.H.V.); lythanhtruonggiang@gmail.com (L.T.T.G.); jongseong@pusan.ac.kr (J.S.H.); jsyun14@hanmail.net (J.Y.); 2 Laboratory for Vascular Medicine and Stem Cell Biology, Department of Physiology, School of Medicine, Pusan National University, Yangsan 50612, Korea; 3 Korea Institute of Toxicology, Dajeon 34114, Korea; 4 Department of Physiology, School of Medicine, Pusan National University, Yangsan 50612, Korea; 5 Research Institute of Convergence Biomedical Science and Technology, Pusan National University Yangsan Hospital, Yangsan 50612, Korea

**Keywords:** CHIR99021, GSK-3β, EPC, mTOR, lysosome, autophagy, senescence

## Abstract

Endothelial progenitor cells (EPCs) are specialized cells in circulating blood, well known for their ability to form new vascular structures. Aging and various ailments such as diabetes, atherosclerosis and cardiovascular disease make EPCs vulnerable to decreasing in number, which affects their migration, proliferation and angiogenesis. Myocardial ischemia is also linked to a reduced number of EPCs and their endothelial functional role, which hinders proper blood circulation to the myocardium. The current study shows that an aminopyrimidine derivative compound (CHIR99021) induces the inhibition of GSK-3β in cultured late EPCs. GSK-3β inhibition subsequently inhibits mTOR by blocking the phosphorylation of TSC2 and lysosomal localization of mTOR. Furthermore, suppression of GSK-3β activity considerably increased lysosomal activation and autophagy. The activation of lysosomes and autophagy by GSK-3β inhibition not only prevented replicative senescence of the late EPCs but also directed their migration, proliferation and angiogenesis. To conclude, our results demonstrate that lysosome activation and autophagy play a crucial role in blocking the replicative senescence of EPCs and in increasing their endothelial function. Thus, the findings provide an insight towards the treatment of ischemia-associated cardiovascular diseases based on the role of late EPCs.

## 1. Introduction

Myocardial ischemia is initiated by an inadequate blood supply to the myocardium which leads to a reduced supply of oxygen to the heart muscles. A sudden and severe blockade to the myocardial artery can lead to heart attack, which is one of the main causes of morbidity and mortality worldwide. EPCs in the circulating blood mark the inauguration of a new era in the field of medicine due to their enormous potential in various fields including regeneration, making them a potential source of new cell formation, migration and angiogenesis [1]. Various studies have documented that EPCs isolated from the mononuclear fraction of peripheral or umbilical cord blood participate in endothelial repair and angiogenesis by differentiation into endothelial cells. The precise definition of EPC remains unclear, which has led to the identification and isolation of three types of EPCs based on their phenotypic and functional characteristics using in vitro culture assays: (1) endothelial colony forming cells (ECFC or late EPC), (2) circulating angiogenic cells (CAC or early EPC) and (3) colony forming unit-Hill (CFU-Hill) cells. [1,2,3]. It has been suggested that a lower amount of EPCs has been associated with aging as well as a wide range of ailments such as diabetes, atherosclerosis and cardiovascular disease [4,5,6]. Indeed, an increased number of EPCs is directly proportional to a low risk of ischemia-associated cardiovascular diseases. Therefore, increasing EPC numbers, differentiation and angiogenesis is essential for the treatment of ischemia and its accompanying diseases.

Glycogen synthase kinase-3 (GSK-3) is a ubiquitously expressed serine/threonine kinase enzyme, which regulates glycogen synthesis in response to insulin and several other growth factors. GSK-3α and GSK-3β are isoforms of GSK-3, which is direct target of the PKB/AKT signaling pathway. The phosphorylation of GSK-3 by AKT at serine 21 in GSK-3α or serine 9 in GSK-3β inhibits the kinase activity of GSK-3 [7,8]. GSK-3 is implicated in several different cellular processes including metabolism, protein synthesis, cell fate, proliferation and survival [7]. GSK-3β is one of the disruptive factors of Wnt signaling, the stimulation of Wnt by GSK-3β inhibition chiefly regulates cell proliferation, differentiation and apoptosis by beta-catenin-dependent or beta-catenin-independent pathways [9]. GSK-3β inhibition-induced Wnt signaling is vital to promote CD133^+^, CXCR4 and CD117-mediated wound healing and to improve angiogenesis against ischemic reperfusion injury in diabetic mice [2,10,11].

Beta-catenin-independent Wnt activation is interconnected with TSC2 (tuberous sclerosis complex) and TOR (target of rapamycin) signaling. TSC2, a tumor suppressor gene, controls the mammalian target of rapamycin complex 1 (mTORC1) activity. GSK-3β can phosphorylate TSC2, and phosphorylation of TSC2 further translocates from the lysosomal surface and stimulates mTOR1 by way of the GTPase-activating protein Rheb [12,13]. GSK-3β is a multi-functional enzyme blocking the lysosome activation and autophagy via mTOR [14,15]. Overactivation of GSK-3β and mTOR1 has been implicated in aging and its associated diseases.

A decline in the lysosome-associated autophagic process escalates damaged protein accumulation, which is directly responsible for cellular senescence and ageing. Intensifying the lysosomal biogenesis and autophagic degradation by deactivating the GSK-3β will continually advance protein synthesis, cell differentiation and growth and prevent cell senescence [15,16,17]. The accumulation and expression of GSK-3β is lethal to various types of cells which induce senescence and apoptosis [18,19]. Cellular senescence is defined as a condition in which a cell permanently loses the ability to proliferate. Prolonged senescence is a complex process that indicates changes in tissue structure and a decline in regenerative capacity [20,21]. Several different pathological and physiological stresses activate senescence by expressing p16^INK^, p21 and p27 [22]. The link between autophagy and senescence is an important concern in the field of ageing research but is still unknown.

Small molecule inhibitors are emerging as important players in both the regulation of stem cell fate and reprogramming. An aminopyrimidine derivative compound CHIR99021 is known for its selective inhibition of the GSK-3β enzyme [23]. Previous studies have documented that the treatment of human pluripotent stem cells with CHIR99021 results in increased functional endothelial cells and their progenitors’ growth and differentiation [24]. Additionally, CHIR99021 not only influences the Wnt/β-catenin pathway but also modifies various other signaling pathways such as TGF-β, Notch and MAPK [25]. However, downstream targets of CHIR99021 are not fully reported. In the present study, we show that CHIR99021-induced GSK-3β inhibition suppresses mTOR, which subsequently augments lysosomal activation and autophagy and prevents replicative senescence of late EPCs. We further identified that lysosome activation and autophagy by GSK-3 inhibition expands the proliferation, migration and angiogenesis of late EPCs.

## 2. Results

### 2.1. GSK-3β Inhibition by CHIR99021 Modulates Late EPCs Functional Activities

**Figure 1:**
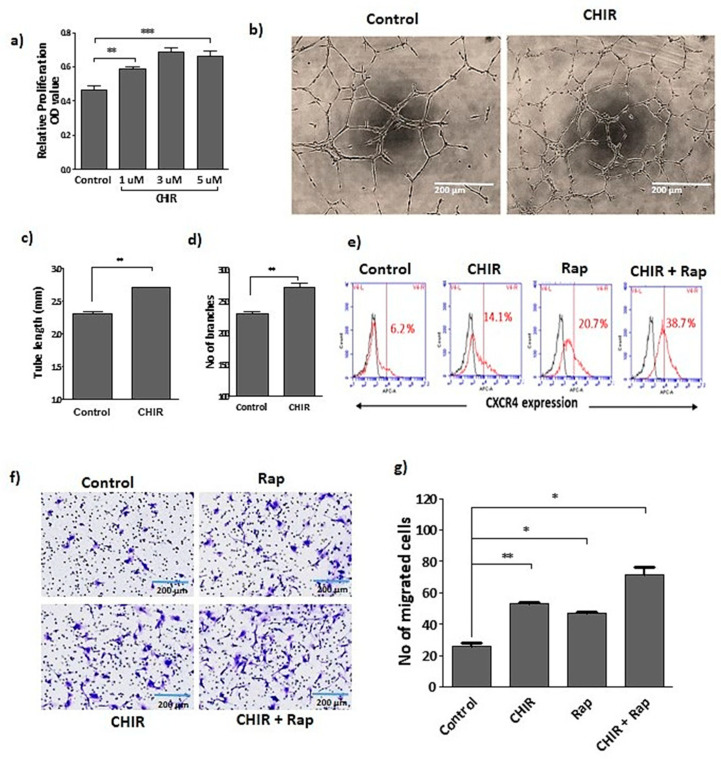
Stimulation of CHIR99021 established the late EPCs functional activity. (**a**) Cells were treated with CHIR99021 at different doses (1, 3, 5 uM) for 24 h, and proliferation was assessed by using wst-1. (**b**) Growth factor reduced Matrigel used for tube formation assay. The vessel structure was visualized using a light microscopy. Tube length and branches were quantified using ImageJ. (**c**,**d**) Quantification of total tube length and number of branches. (**e**) The treatment with CHIR99021 (3 uM) alone or co-treated with rapamycin (20 nM) for 24 h in late EPCs. Fluorescence activated cell sorting was performed by using non-stained cells as a negative control. The fraction of positively stained cells was determined by comparison with non-stained cells. The fraction of positively stained cells is indicated by the positive peaks (red lines indicate cells stained with each antibody, and black lines indicate the negative control). (**f**) Transwell migration assay was performed by seeding cells in the upper inserts of the transwell chambers, whereas medium containing the drug was loaded into lower chambers of the plate. The cells were incubated for up to 6 h, and the number of migrated cells was counted in three random fields for each membrane filter (20× magnification) under microscope. (**g**) Quantification of migrated cells. Data are presented as mean ± standard error of the mean (SEM). The results are considered statistically significant at * *p*< 0.05; ** *p*< 0.01; *** *p* < 0.001 when compared to untreated groups. (Rap-Rapamycin).

Late EPCs treated with CHIR99021 showed a dose-dependent increase in the proliferation rate (Figure 1a). Treatment of CHIR99021 significantly improved the number of branches and total tube length formation, CXCR4 expression and migration in the late EPCs (Figure 1b–g). We observed that inhibition of GSK-3β by CHIR99021 treatment enhanced the late EPCs functional activities, such as proliferation, migration and angiogenesis.

### 2.2. GSK-3β Inhibition by Using CHIR99021 Deregulates mTOR via Rheb Inhibition

**Figure 2:**
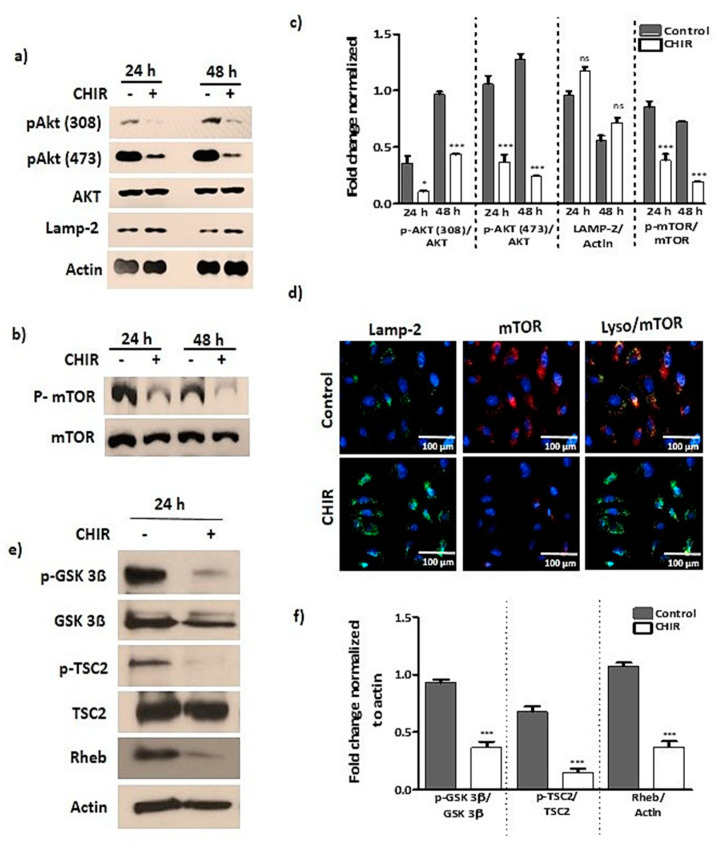
GSK-3β inactivation by CHIR99021 treatment deregulates AKT and mTOR signaling. (**a**–**c**) Cells were treated with CHIR99021 (3 uM) for 24, 48 h, then Western blot was performed to detect the phosphorylation and total form of AKT, mTOR, Rheb, LAMP-2 and actin (taken as loading control). (**d**) The cells were selectively treated with CHIR99021 (3 uM) for 24 h, then subsequently immunostaining was performed to evaluate the lysosomal localization of mTOR. (**e**,**f**) Followed by 24 h of CHIR99021 (3 uM) treatment, whole cell lysate fractions were isolated, then Western blot was performed to detect phosphorylation and total form of GSK-3β, TSC2, Rheb, and actin taken as a loading control. Data are presented as mean ± standard error of the mean (SEM). The results are considered statistically significant at * *p*< 0.05; *** *p* < 0.001 when compared to untreated group, and ns (no significant).

We observed that when the late EPCs were treated with CHIR99021, there was significant reduction in the phosphorylation of Akt in a time-dependent manner (Figure 2a,c). CHIR99021 treatment also significantly down regulated the mTORC, TSC2 and Rheb levels (Figure 2b,c,e,f). We noted that the late EPCs treated with CHIR99021 reduced the lysosomal accumulation of mTOR (Figure 2d). Our data suggest that CHIR99021-induced GSK-3β inhibition deregulates mTOR and its downstream signaling through TSC2/Rheb.

### 2.3. CHIR99021-Induced GSK-3β Inhibition Enhances Lysosome Activation and Autophagy

**Figure 3:**
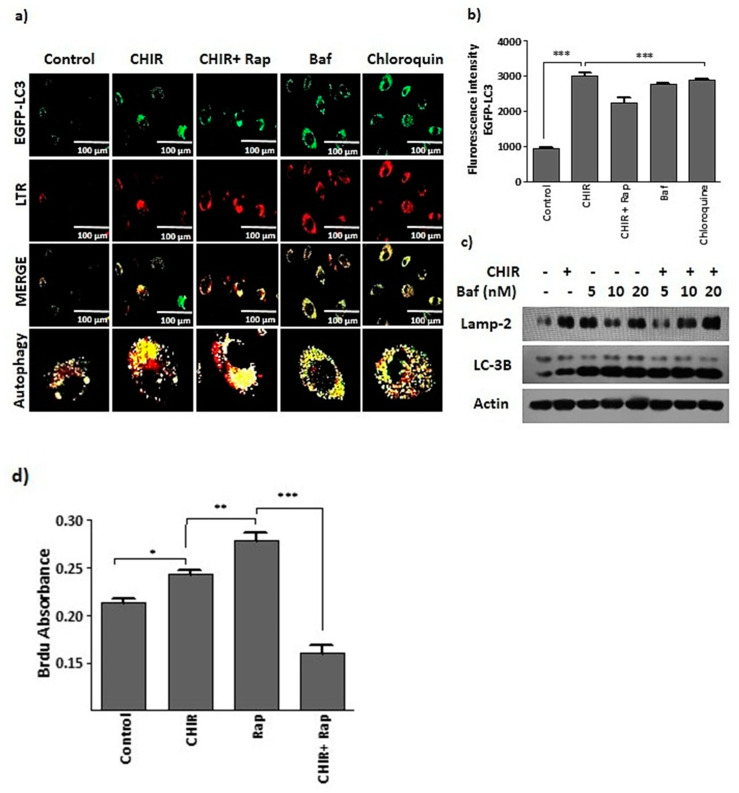
The suppression of GSK-3β using CHIR99021 upregulates lysosome activation and autophagy. (**a**,**b**) EPCs were transfected with GFP-LC3 plasmid using Lipofectamine 3000 reagent. Then, cells were selectively treated with CHIR99021 (3 uM), rapamycin (20 nM), lysosomal blocker bafilomycin A1 (5 nM) and autophagy blocker chloroquine (20 uM) for 4 h. Next, cells were co-stained with lysotracker (200 nM) for 1 h. GFP-LC3 expression was captured using a 40× objective lens on a Lion Heart FX automated microscope. Scale bar = 100 μM. (**c**) The cells were treated with CHIR99021 (3 uM) alone or co-treated with bafilomycin A1 (5, 10, 20 nM) for 4 h, then Western blots were generated to detect the expression of lysosomal marker proteins LAMP2 and autophagy marker protein LC-3B. Actin was taken as a loading control. (**d**) The cells were selectively treated with CHIR99021 (3 uM) for 24 h, rapamycin (20 nM) and bafilomycin A1 (5 nM) for 1 h. BrdU incorporation was absorbed at 450 nm to assess the proliferation. Data are presented as mean ± standard error of the mean (SEM). The results are considered statistically significant at * *p*< 0.05; ** *p*< 0.01; *** *p* < 0.001 when compared to untreated groups, and ns (no significant). (Baf—bafilomycin A1, Rap—Rapamycin).

To perceive the connection between GSK-3β inhibition-induced lysosome activation and autophagy, cells that were selectively treated with CHIR99021 had extensively increased GFP-LC3 and lysotracker expressions (Figure 3a). Subsequent experiments showed that treatment with CHIR99021 upregulated the expression of LAMP2 (lysosomal marker protein) and LC-3B (autophagy marker protein). Moreover, co-treatment of cells with bafilomycin A1 (lysosomal activation blocker) and chloroquine (an autophagy blocker) consistently blocked lysosome activation and autophagy (Figure 3a–c). Lysosome activation and autophagy were assessed through GSK-3β inhibition by selective treatment with CHIR99021 alone or co-treatment with rapamycin, a well-known mTOR inhibitor and autophagy activator, which as expected increased GFP-LC3 expression and BrdU absorption (Figure 3d). In contrast, BrdU absorption was dramatically reduced by lysosomal blocker bafilomycin A1 (Figure 3d). We found that treatment with CHIR99021-induced GSK-3β inhibition subsequently improved lysosome activation and autophagy.

### 2.4. Lysosome Activation and Autophagy by the Way of GSK-3β Inhibition Augments Late EPCs Functional Activity

**Figure 4:**
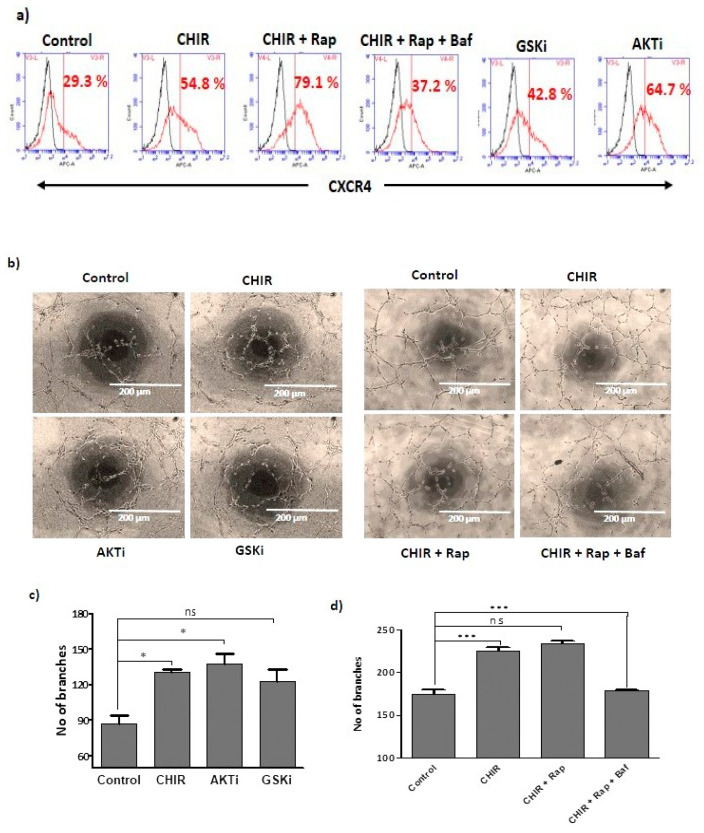
Lysosome activation and autophagy by CHIR99021 expands endothelial functional activity of late EPCs: (**a**) Cells were single treated with CHIR99021 (3 uM), GSK-3β-specific inhibitor (3 uM) for 24 h and AKT 1/2 inhibitor (3 uM) for 1 h, then subsequently co-treated with rapamycin (20 nM) and bafilomycin A1 (5 nM) for 1 h. FACS was performed for gating the population of non-stained cells as a negative control. The fraction of positively stained cells was determined by comparison with non-stained cells. The fraction of positively stained cells is indicated by the positive peaks (red line indicates cells stained with each antibody, and black lines indicate the negative control). (**b**) Endothelial functional activity of EPCs was established using vascular network formation. Cells were treated with CHIR99021 (3 uM), AKT 1/2 inhibitor (3 μM) and GSK-3β inhibitor (3 uM) either in the presence or absence of rapamycin (20 nM) and bafilomycin A1 (5 nM) for 6 h. The vascular structure was visualized using a light microscopy; the branches were quantified using ImageJ. (**c**,**d**) The number of branches were also quantified. Data are presented as mean ± standard error of the mean (SEM). The results are considered statistically significant at * *p* < 0.05; *** *p* < 0.001 when compared to untreated groups and ns (no significant. (Baf—bafilomycin A1, Rap—Rapamycin, AKTi—AKT inhibitor, GSKi—GSK inhibitor).

To understand the importance of lysosome activation and autophagy for late EPCs functional activity, we treated the late EPCs with CHIR99021, GSK-3β and AKT inhibitors alone. These drugs as single agents steadily improved the EPCs specific surface marker expression of CXCR4 (Figure 4a) and number of branches (Figure 4b–d). Interestingly, late EPCs treated with rapamycin alone or in combination with CHIR99021 dramatically increased CXCR4 expression, the number of branches. (Figure 4a,b,d) and the number of migrated cells (Figure 1f,g) in comparison to the control. Whereas, cells co-treated with bafilomycin A1 significantly blocked the CHIR99021-induced CXCR4 expression (Figure 4a, and the number of branches (Figure 4d). Together, our findings suggest that CHIR99021-induced lysosome activation and autophagy improves the angiogenic potential of late EPCs.

### 2.5. Lysosome Activation and Autophagy Prevent Late EPCs Senescence

**Figure 5:**
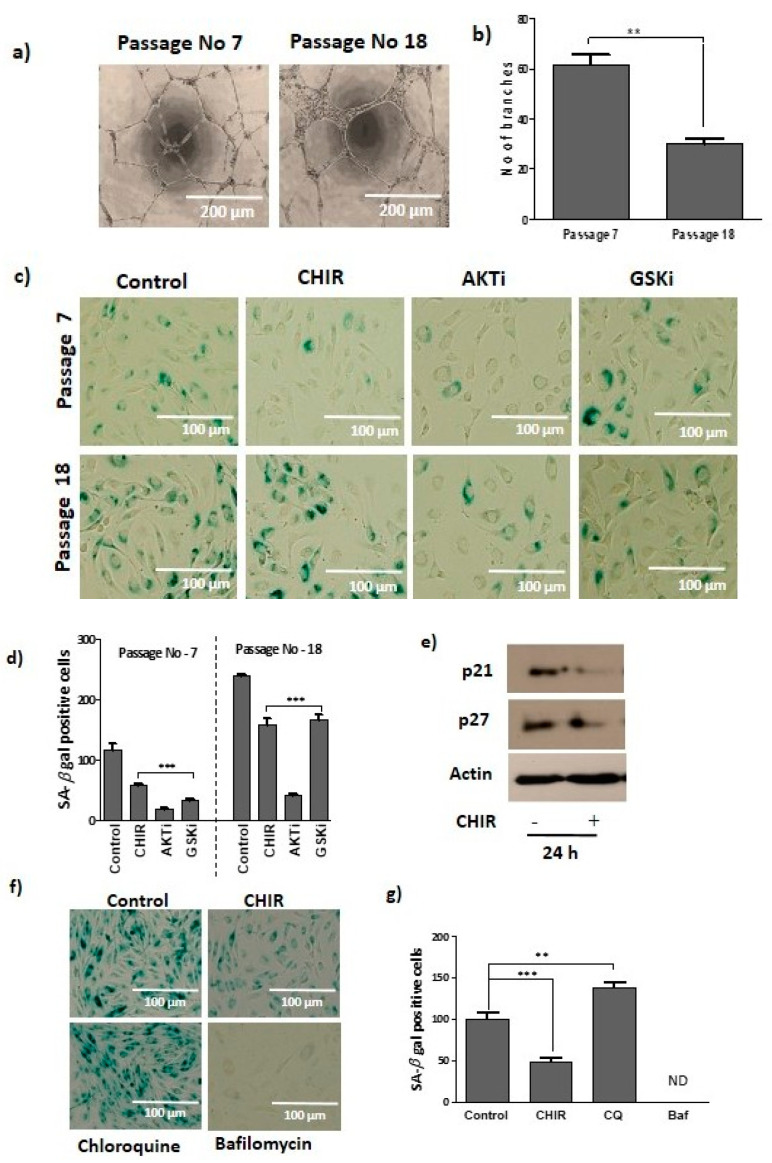
Late EPCs senescence repressed by lysosome activation and autophagy. (**a**) The formation of vascular structure was compared with young (p-7) and old (p-18) passage numbers of late EPCs. (**b**) The number of branches was quantified by young (p-7) and old (p-18) cells. (**c**) The cells were treated with CHIR99021 (3 uM) and GSK-3β inhibitor (3 uM) for 24 h, and AKT 1/2 inhibitor (3 uM) for 1h, at different passages (p-7, p-18). Following this, the cells were stained to detect senescence by β-galactosidase according to the manufacturers’ instructions. (**d**) The senescence-positive cells were quantified. (**e**) Cells were stimulated with CHIR99021 (3 uM) for 24 h, and senescence marker proteins p21 and p27 and loading control actin expression was observed using Western blots. (**f**) After selective treatment with CHIR99021 (3 uM), chloroquine (20 uM) and bafilomycin A1 (5 nM), cells were stained with β-galactosidase. (**g**) The senescence-positive cells were captured and quantified. Data are presented as mean ± standard error of the mean (SEM). The results are considered statistically significant at ** *p*< 0.01; *** *p* < 0.001 when compared to untreated groups. (Baf—bafilomycin A1, CQ—chloroquine).

To evaluate the progression of senescence, late EPCs were stained with beta-gal as marker of senescent cells. Beta-gal staining was significantly different between passage 7 and passage 18 EPCs (Figure 5c,d). To further ensure that cellular senescence diminished the late EPCs functional activity, a tube formation assay was performed. The assay illustrated that total tube length and number of branches declined in the high-passage-number EPCs (p-18) when compared to low-passage-number EPCs (p-7) (Figure 5a,b). Therefore, we aimed to understand whether CHIR99021-induced GSK-3β inhibition would prevent late EPCs senescence.

Late EPCs treated with CHIR99021 for 24 h noticeably reduced the senescence marker proteins such as p21 and p27 (Figure 5e). Additionally, cells singly treated with CHIR99021, AKT and GSK-3β considerably reduced the beta-gal-stained positive cells, irrespective of the passage number in comparison to the vehicle treated control cells (Figure 5c,d). Treatment with CHIR99021 significantly improved the number of branches in low-passage (p-7) and also in high-passage-number EPCs (p-18) (Supplementary Figure S2b,c). In addition, blocking the autophagic process by chloroquine (autophagy blocker) increased the beta-gal-positive cells (senescence) more than the vehicle-treated cells. Beta-gal-positive cells were not detected in bafilomycin A1-treated cells (Figure 5f–g). Collectively, these results suggest that CHIR99021-exposed lysosome activation and autophagy is necessary to prevent late EPCs senescence.

### 2.6. GSK-3β Inhibition by CHIR99021 Deregulates mTOR Signaling by Rheb-Dependent and Beta-Catenin-Independent signaling

**Figure 6:**
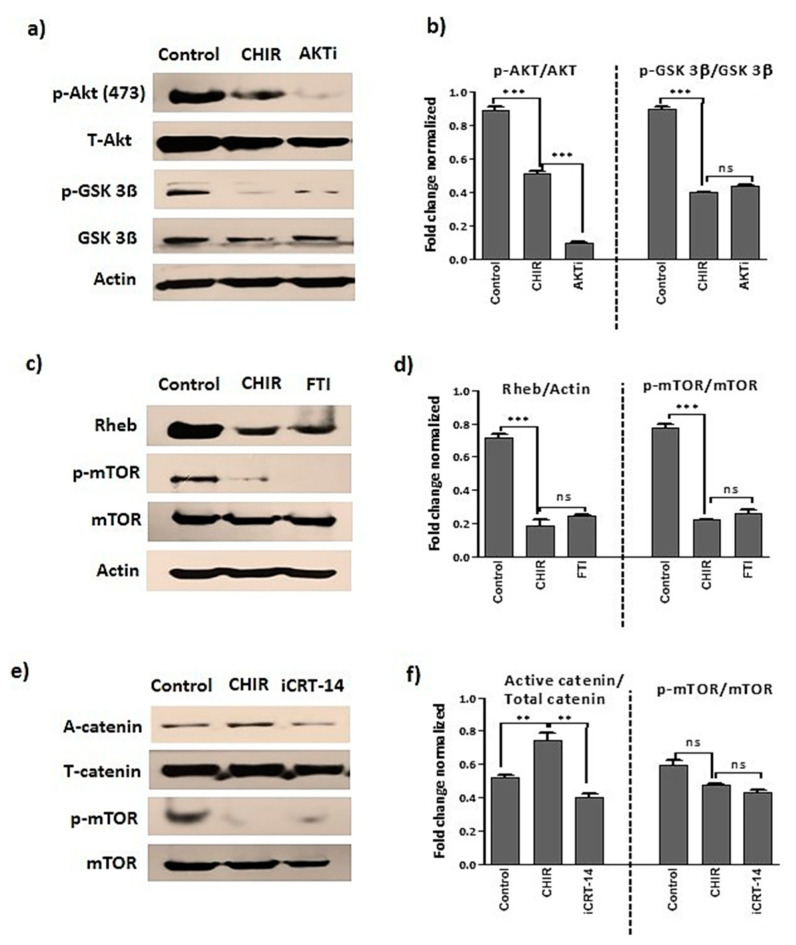
GSK-3β inactivation deregulates mTOR through Rheb signaling. (**a**–**f**) Late EPCs stimulated with CHIR99021 (3 μM), AKT 1/2 inhibitor (3 μM), Rheb inhibitor (10μM) and beta-catenin inhibitor (10μM) for 24 h. Then, Western blot was performed to evaluate the protein level expression of p-AKT, AKT, active catenin, total catenin, p-GSK-3β, GSK-3β, Rheb, LAMP2, p-mTOR, mTOR and loading control actin. Data are presented as mean ± standard error of the mean (SEM). The results are considered statistically significant at ** *p*< 0.01; *** *p* < 0.001 when compared to untreated groups, and ns (non-significant). (AKTi—AKT inhibitor, FTI—Farnesyltransferase, a Rheb inhibitor, iCRT-14, beta-catenin inhibitor).

In order to assess the link between CHIR99021-induced blockade of AKT and GSK-3β phosphorylation, late EPCs were treated with AKT 1/2 inhibitor or CHIR99021 for 24 h. The cells treated with either AKT 1/2 inhibitor or CHIR99021 moderately inhibited the phosphorylated form of AKT. Moreover, in co-treatment conditions, no significant changes occurred in the total form of AKT. Next, the phosphorylated and total form of GSK-3β were reliably decreased in CHIR99021 treatment as compared to AKT 1/2 inhibitor (Figure 6a,b). Further, AKT 1/2 inhibitor significantly increased CXCR4 expression as compared to cells treated with CHIR99021 and GSK-3β-specific inhibitors (Figure 4a). In addition, senescence-positive beta-gal assay confirmed, the inhibition of AKT by AKT 1/2 inhibitor extensively prevented senescence-positive cells in comparison to CHIR99021 and GSK-3β-specific inhibitor-treated cells (Figure 5c,d). Further, to confirm whether CHIR99021-induced mTOR inhibition was Rheb dependent or independent, cells were treated with CHIR99021 and a Rheb inhibitor (Farnesyltransferase inhibitor). In this study, CHIR99021 and Rheb treatment correspondingly inhibited the expressions of Rheb and p-mTOR (Figure 6c,d). Furthermore, to identify whether the CHIR99021-induced mTOR deregulation was beta-catenin dependent or independent, EPCs were treated with CHIR99021 and a beta-catenin inhibitor (iCRT-14). As a result, the stimulation of CHIR99021 alone upregulated the expression of active catenin and co-treatment with iCRT-14 downregulated the active beta-catenin expressions. Additionally, stimulation of CHIR99021 alone and co-treatment with iCRT-14 consistently deactivated the p-mTOR expressions (Figure 6e,f), (Supplementary Figure S3a,b).

## 3. Discussion

Ischemia is the lack of oxygen and nutrient supply to part of the body leading to irreversible damage to the affected tissue. Associated with it is a severe condition called myocardial ischemia, which causes high morbidity and mortality worldwide [26]. It is a known fact that tissue ischemia can be attenuated by stimulating EPCs proliferation, mobilization and angiogenesis at the injured sites, which displays the regenerative potential in ischemic tissues [27,28]. Over time, cell-based therapy has emerged as a promising therapeutic tool for the treatment of ischemia-related cardiovascular disease. Hence, expanding EPCs growth, migration and angiogenesis is considered to be a vital approach towards the treatment of ischemic cardiovascular disease. Isolation of EPCs from umbilical cord blood, bone marrow or circulation blood is characterized by Di-acetylated LDL and Ulex-1 binding or surface level marker expression of CD34, CXCR4, c-KIT, VEGFR-2 and CD-133 [2,10,11]. CXCR4/SDF-1, a member of the CXC family of chemokines, plays an important role in EPCs proliferation and migration. Relatedly, our study clearly indicates that CHIR99021-induced GSK-3β inactivation augments not only the proliferation and angiogenesis of late EPCs but also, as compared to all other EPCs surface markers, CXCR4 was dramatically increased in addition to the migration of late EPCs.

Glycogen synthase kinase is a serine/threonine kinase which consists of GSK-3α and GSK-3β. GSK-3β controls a large number of cellular processes including proliferation, differentiation, angiogenesis, apoptosis and autophagy [29,30]. The activation of GSK-3β is also associated with ageing, diabetes, Alzheimer’s disease and ischemia-associated vascular complications [19,31]. GSK-3β is inactivated by a variety of extracellular stimuli including insulin and growth factors [32]. CHIR99021, a well-known inhibitor of GSK-3β, nonspecifically and consistently inhibits the phosphorylation of AKT. Meanwhile, AKT, also known as protein kinase B, is an upstream of GSK-3β identified as phosphorylating GSK-3β at Ser-9 to inactive its kinase activity. In the present study we found a contradictory effect: CHIR99021 not only inhibited the phosphorylated form of GSK-3β but also nonspecifically suppressed the phosphorylated form of AKT. The connection between the CHIR99021-induced inhibition of phosphorylated AKT and GSK-3β in late EPCs is unknown.

AKT, a serine/threonine protein kinase activated by insulin growth factors, ultimately regulates various cellular processes including cell proliferation, migration, apoptosis, angiogenesis and autophagy [13,33,34,35,36,37]. In the present study, the treatment of AKT 1/2 inhibitor or CHIR99021 moderately inhibited the phosphorylated form of AKT; moreover, in both treatments no significant changes were found in the total form of AKT. Further, it is established that AKT inhibition limits the proliferation (data not shown) of late EPCs, and unexpectedly we found that inhibition of AKT enormously increases the EPCs’ specific surface marker expression of CXCR4 and prevents cellular senescence marker expressions as compared to CHIR99021 treatment. However, the actual mechanism behind the AKT inhibition-induced CXCR4 expression and delaying of late EPCs senescence is unknown.

The regulation and deregulation of mTOR is involved in a wide range of cellular functions including cell growth, metabolism, lysosome activation and autophagy [38,39,40,41,42,43]. AKT phosphorylates several upstream and downstream targets, including GSK-3/TSC2/mTOR signaling [44]. mTOR is a direct target of tuberous sclerosis complex (TSC), which consists of TSC1 and TSC2 [45]. The phosphorylation of TSC2 by AKT and GSK-3β represses the GAP activity of the TSC complex, allowing Rheb to accumulate in the lysosome surface, which subsequently activates mTOR1 and mTOR2 [12,13,16,45,46]. In the present study, whole-cell lysate suggested the fact that the inactivation of GSK-3β subsequently inhibits the phosphorylation of TSC2 and mTOR signals via Rheb inhibition. Further, lysosomal accumulation of Rheb or mTOR directly or indirectly regulates mTOR activity. Our immune staining data unveiled that GSK-3β inhibition consistently suppresses the lysosomal localization of mTOR.

The activation of Wnt signaling plays an important role in several different cellular processes through beta-catenin-dependent or beta-catenin-independent pathways. Beta-catenin-dependent Wnt stimulation is regulated by disrupting the beta-catenin destruction complex, which augments beta-catenin expression [9,47,48]. On the other hand, beta-catenin-independent Wnt stimulation is TSC2/TOR dependent [12,49]. In our study, treatment with CHIR99021 enhances the expression of active beta-catenin and decreases the Rheb and phosphorylated mTOR. To find whether beta-catenin activation has any role in the deactivation of Rheb and phosphorylated mTOR, we further inhibited the beta-catenin in late EPCs and related the expression of Rheb and phosphorylated mTOR in both conditions. Unexpectedly, we found that inhibition of Rheb and phosphorylated mTOR expressions were beta-catenin independently regulated by CHIR99021 in late EPCs.

**Figure 7:**
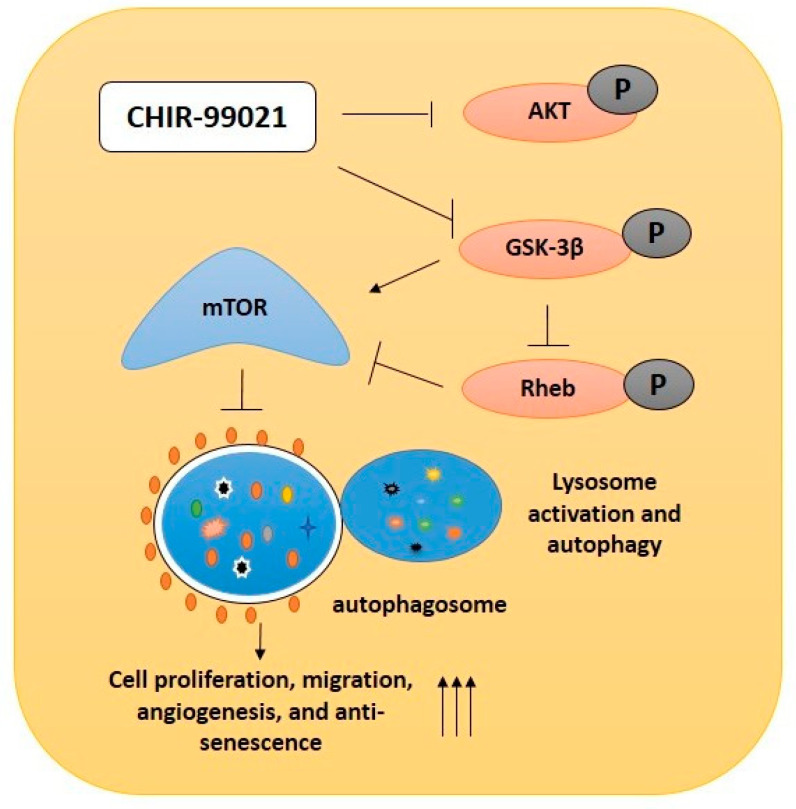
Graphical representation. Schematic representation of GSK-3β inhibition in late EPCs using CHIR99021. The CHIR99021-induced GSK-3β inactivation nonspecifically inhibited the phosphorylated form of AKT. Inhibition of GSK-3β subsequently inhibited Rheb activity and suppressed mTOR activity. In addition, CHIR99021 treatment upregulated lysosome activation and autophagy, which further prevents cellular senescence and thereby increases bioactivity such as cell proliferation, migration and angiogenesis in late EPCs.

Autophagy is a self-degradation pathway which is known to eliminate damaged proteins and sub-cellular organelles. Insufficient activation of lysosome and autophagy enriches the damaged protein accumulation, implicating aging and age-related vascular complications [17]. The genetic and pharmacological activation of autophagy expands the life span of different model organisms [50,51]. GSK-3 is the central regulator of lysosome activation and autophagy by mTOR, controlling protein synthesis, cell size and growth [19,52]. Recently, Leeman et al. and others reported that lysosomal activation and autophagy by mTOR inhibition improved quiescent stem cell activation and differentiation [17,53,54,55]. Currently, our data also suggest that cells that were incubated with CHIR99021 steadily activate lysosome and autophagy by the inhibition of Rheb and mTOR. Further, the activation of lysosome and autophagy increases late EPCs proliferation, migration and vascular structure formations (Figure 7). Cellular senescence is one of the greatest risk factors, which is caused by many pathological conditions leading to the functional decline of regenerative potential. In the present study, lysosome activation and autophagy by CHIR99021 stimulation consistently recover the replicative senescence of late EPCs. In addition, treatment of lysosomal and autophagy blockers reverts the CHIR99021-induced EPCs proliferation, vascular formation and cellular senescence.

## 4. Materials and Methods

### 4.1. Isolation of Late EPCs and Cell Culture

Human umbilical cord blood (HUCB) was collected from healthy volunteers according to the protocol approved by the Institutional Review Board of Pusan National University, Yangsan Hospital, South Korea (Approval No. PNUYH-05-2017-053). Mononuclear cells (MNCs) were isolated from human umbilical cord blood by density gradient centrifuge using Ficoll (GE Healthcare, Duckinghanshire, UK). HUCB-derived MNCs were maintained in an EGM-2 bullet kit system (Lonza, Walkerville, MD, USA) containing endothelial basal medium 2 (EBM-2), 5% fetal bovine serum (FBS), human vascular endothelial growth factor (hVEGF), human basic fibroblast growth factor (b-FGF), human epidermal growth factor (EGF), human insulin-like growth factor-1 (IGF-1), ascorbic acid and GA-1000. Culturing MNCs an EGM-2 media formulation maintain outstanding EPC morphology and functions. Cells were seeded on 1% gelatin (Sigma, St. Louis, MO, USA)-coated plates and incubated at 37 °C in 5% CO_2._ After 5 days, non-adherent cells were discarded, and fresh culture medium was added. The adherent cells were assessed for the ability to uptake Dil acetylated-LDL or binding of fluorescently labelled Ulex europaeus agglutin 1 (Ulex-1) plant lectin. The adherent cells that took up Dil acetylated-LDL or bound Ulex europaeus agglutin 1 plant lectin were considered late EPCs (Supplementary Figure S1) [56]. These adherent cells were further confirmed by expression of EPCs surface markers by flow cytometry analysis, and cultures were maintained for 10–14 days until the development of colonies. The culture medium was changed daily, and colonies were re-plated and cultured for further studies.

### 4.2. Flow Cytometer Analysis

Cultured late EPCs were identified and characterized by flow cytometry using hematopoietic stem cells and angiogenesis markers CD34, CXCR4, c-Kit, VEGFR2 purchased from BD Pharmingen (Franklin Lake, NJ, USA). For FACS analysis, the harvested cells were washed with 1× PBS, and cells were stained with appropriate antibody diluted with FACS buffer for 30 min at 4 °C and washed with FACS buffer three times. Afterward, flow cytometry analysis was carried out by fluorescence-activated cell sorting (FACS; BD FACS canto 2, San Jose, CA, USA). Fluorescence-activated cell sorting was performed using non-stained cells as a negative control. The fraction of positively stained cells was determined by comparison with non-stained cells. The fraction of positively stained cells is indicated by the positive peaks (red line indicate cells stained with each antibodies, and black lines indicate the negative control).

### 4.3. Cell Counting Kit-8 (CCK-8) Assay

Cell proliferation was detected using the D-Plus cell counting kit (CCK-8) according to the manufacturers’ instructions (Dongin Biotech, Seoul, Korea). Cells were seeded into 96-well plates. The cells were incubated overnight at 37 °C in a 5% CO_2_ incubator, and then cells were selectively treated with CHIR 99021 (Biovision, CA, USA) for 24 and 48 h. Next, cells were treated with (10 μL/well) WST-1 for 1 h in the dark at 37 °C in a 5% CO_2_ incubator. The absorbance of each well was measured at 450 nm using a Tecan (XFluor, Zurich, Switzerland) microplate reader. Each experiment was repeated five times.

### 4.4. BrdU Incorporation Assay

Cells were selectively treated with CHIR 99021 (3 uM), rapamycin 20 nM, and bafilomycin A1 (5 nM) for 24 h. Then BrdU incorporation into the newly synthesized DNA was used for detection according to the manufacturers’ instructions from Roche (Mannheim, Germany). The absorbance of each well was measured at 450 nm using a Tecan (XFluor, Mannedorf, Switzerland) microplate reader. Each experiment was repeated five times.

### 4.5. Transwell Migration Assay

Cell migration assays were performed using 24-transwell migration chamber inserts (8 μm pore size; Columbia, WA, USA). Late EPCs (2 × 10^4^) were seeded on the upper inserts, and lower chambers were filled with medium, either with or without CHIR-99021 (3 μM) and rapamycin 20 nM; then, the cells were incubated for 6 h at 37 °C. Then, the cells in the upper membrane were removed with a cotton swab, and cells which migrated to the lower surface were fixed with 4% paraformaldehyde (PFA) and stained with 0.5% crystal violet in 25% methanol for 30 min. The number of migrated cells was counted under a microscope (magnification, 200×) by randomly selecting three fields per filter.

### 4.6. Tube Formation Assay

Growth factor reduced Matrigel (BD Bioscience, San Jose, CA, USA) was used for the experiment. GFR-Matrigel was thawed at 4 °C a day before the experiment, and then 96 well plates were coated with Matrigel (60 μL/well) and polymerized for 30–45 min at 37 °C. Cells were seeded on Matrigel-coated wells at a density of 6 × 10^3^ cells/well in the full medium, selectively treated with CHIR 99021 (GSK-3 β inhibitor), AKT 1/2 inhibitor, GSK-3β inhibitor (Specific), bafilomycin A1 (Lysosome pH sensitive blocker) and chloroquine (Autophagy blocker) for 6 h at 37 °C in CO_2_ incubator. Next, tube length and branch formation were measured by counting the number of branches and measuring their length under microscopic at 20× magnification.

### 4.7. Immunoblots

Total cell lysates were extracted using PRO-PREP protein extraction buffer (Intron biotechnology, Seoul, Korea), and protein concentration was determined by a BCA assay kit (Thermo Scientific, Rockford, IL, USA). Protein samples were separated with SDS-PAGE and transferred onto PVDF membranes (Millipore, Billerica, MA, USA). The membranes were blocked with 5% skim milk for 1 h and incubated with primary antibodies, active beta-catenin, p-AKT (Ser 473, 308), AKT, p-mTOR (Ser 2448), mTOR, Raptor, Rictor (Cell Signaling Technology, Boston, MA, USA), p-TSC2,TSC2, p-GSK-3β, GSK-3β, LAMP1, LAMP2, Rheb, tubulin, Beta-Actin (from Santa Cruz Biotechnology, Dallas, Texas USA) and LC-3B (abcam) overnight at 4 °C, followed by incubation with HRP conjugated goat anti-rabbit IgG (Enzo Life Sciences, NY, USA) and goat anti-mouse IgG (Enzo Life Sciences, NY, USA) secondary antibodies at 25 °C for 1 h. Western blots used chemiluminescent detection (Immobilon, Millipore, Burlington, MA, USA).

### 4.8. Immunofluorescence

A total of 5 × 10^4^ cells were seeded in poly-L lysine-coated 6-well plates and then incubated overnight. Post treatment with drugs, the cells were fixed with 3.7% PFA for 10 min at 25 °C. Samples were washed and permeabilized with 0.1% Triton ×-100 in 1× PBS for 10 min and blocked with 1% BSA in (0.3 M glycine) for 1 h, followed by staining with primary antibodies mTOR, LAMP2 (Santa Cruz Biotechnology, CA, USA) overnight at 4 °C in the dark. Post incubation, the slides were washed thrice with PBS, followed by incubation with secondary antibodies (Alexa flour 488, 594, Life science technologies, Carlsbad, CA, USA) for 2 h at room temperature in the dark. Then, the slides were washed and stained with DAPI (Sigma, St. Louis, MO, USA). Slides were covered with a coverslip using prolong anti-fade diamond mountant, and images were captured using a Lion Heart FX automated microscope (Biotek, Winooski, VT, USA) at 40× magnification. Scale bar (100 μm).

### 4.9. Transfections with EGFP-LC3 Plasmid

Late EPCs were transfected with tandem fluorescent-tagged EGFP-LC3 (a gift from Karla Kirkegaard; Add gene, 11546) using Lipofectamine 3000 reagent according to the manufacturers’ protocol. Post 6 h of transfection, the cells were treated with GSK-3β inhibitor and AKT 1/2 inhibitor, bafilomycin A1 and chloroquine. The cells were co-stained with lysotracker and DAPI (Sigma, St. Louis, MO, USA). Slides were covered with a coverslip using prolong anti-fade diamond mountant, and images were captured using a Lion Heart FX automated microscope (Biotek, USA) at 40× magnification. Scale bar (100 μm).

### 4.10. Senescence Associated β-Galactosidase Assay

The senescence-associated β-gal staining is widely used to assess cellular ageing. Late EPCs were exposed to CHIR 99021 (3 μM) for 24 h, AKT 1/2 inhibitor (3 μM), GSK-3β inhibitor, chloroquine (20 μM) and bafilomycin A1 (5 nM) for 1 h. Then, cells were stained with senescence β-galactosidase according to the manufacturers’ instructions (Cell Signaling technology, Boston, MA, USA). Senescence-positive β-gal cells were captured using a 20× objective lens on a Lion Heart FX automated microscope. The senescence-positive cells were further quantified using ImageJ software.

### 4.11. Statistical Analysis

Statistical analyses were performed using GraphPad prism software (v5), and a one-way analysis of variance (ANOVA) was used to assess the differences between experimental and control groups. Data are presented as mean ± standard error of the mean (SEM). The results were considered statistically significant at *p* < 0.05 (*). *p* values less than 0.01 or 0.001 were indicated with ** or ***, respectively. The experimental data presented were an average of three independent experiments.

## 5. Conclusions

Our study revealed that CHIR99021-induced GSK-3β inhibition upregulates lysosomal activation and autophagy. The activation of lysosome and autophagy prevents replicative senescence and improves the bioactivity of late EPCs such as cell proliferation, angiogenesis and migrations.

## Data Availability

The data used to support the findings of this study are included in the article.

## Supplementary Materials

The following are available online at https://www.mdpi.com/article/10.3390/ijms22094796/s1.
