# Correction: Rethineswaran et al. CHIR99021 Augmented the Function of Late Endothelial Progenitor Cells by Preventing Replicative Senescence. *Int. J. Mol. Sci.* 2021, *22*, 4796

**DOI:** 10.3390/ijms232113020

**Published:** 2022-10-27

**Authors:** Vinoth Kumar Rethineswaran, Da Yeon Kim, Yeon-Ju Kim, WoongBi Jang, Seung Taek Ji, Le Thi Hong Van, Ly Thanh Truong Giang, Jong Seong Ha, Jisoo Yun, Jinsup Jung, Sang-Mo Kwon

**Affiliations:** 1 Convergence Stem Cell Research Center, Pusan National University, Yangsan 50612, Korea; 2 Laboratory for Vascular Medicine and Stem Cell Biology, Department of Physiology, School of Medicine, Pusan National University, Yangsan 50612, Korea; 3 Korea Institute of Toxicology, Dajeon 34114, Korea; 4 Department of Physiology, School of Medicine, Pusan National University, Yangsan 50612, Korea; 5 Research Institute of Convergence Biomedical Science and Technology, Pusan National University Yangsan Hospital, Yangsan 50612, Korea

There was an error in representative images of the tube formation in Figure 4b in the original publication [1]. We inadvertently depicted the CHIR-treated image instead of CHIR+RAPA-treated groups in the representative image panel of Figure 4b. The corrected Figure 4b appears below. The authors sincerely apologize for the errors and state that the corrections do not affect the scientific conclusions of the article. The academic editor approved this correction, and the original publication has also been updated.

**Figure 4:**
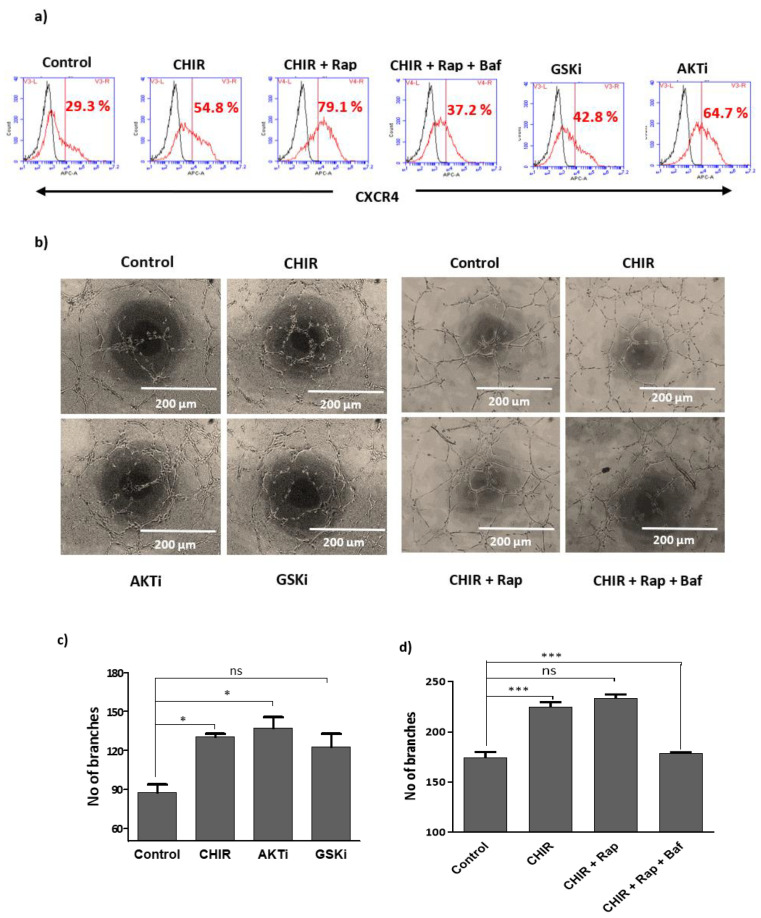
Lysosome activation and autophagy by CHIR99021 expands endothelial functional activity of late EPCs: (**a**) Cells were single treated with CHIR99021 (3 μM), GSK-3β-specific inhibitor (3 μM) for 24 h and AKT 1/2 inhibitor (3 μM) for 1 h, then subsequently co-treated with rapamycin (20 nM) and bafilomycin A1 (5 nM) for 1 h. FACS was performed for gating the population of non-stained cells as a negative control. The fraction of positively stained cells was determined by comparison with non-stained cells. The fraction of positively stained cells is indicated by the positive peaks (red line indicates cells stained with each antibody, and black lines indicate the negative control). (**b**) Endothelial functional activity of EPCs was established using vascular network formation. Cells were treated with CHIR99021 (3 μM), AKT 1/2 inhibitor (3 μM) and GSK-3β inhibitor (3 μM) either in the presence or absence of rapamycin (20 nM) and bafilomycin A1 (5 nM) for 6 h. The vascular structure was visualized using a light microscopy; the branches were quantified using ImageJ. (**c**,**d**) The number of branches were also quantified. Data are presented as mean ± standard error of the mean (SEM). The results are considered statistically significant at * *p* < 0.05; *** *p* < 0.001 when compared to untreated groups and ns (non-significant). (Baf—bafilomycin A1, Rap—Rapamycin, AKTi—AKT inhibitor, GSKi—GSK inhibitor).
