# Supplementary material for: CHIR99021 Augmented the Function of Late Endothelial Progenitor Cells by Preventing Replicative Senescence
Source: Int J Mol Sci. 2021 Apr 30;22(9):4796. doi: 10.3390/ijms22094796 (PMC8124445; doi:10.3390/ijms22094796)
Supplement: Supplementary file 1 [file ijms-22-04796-s001.zip › ijms-1208382-supplementary.pdf]

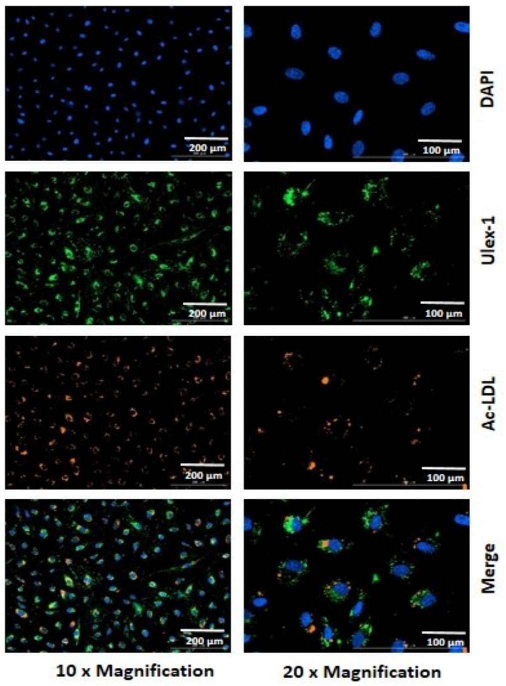

Figure S1. Late EPCs characterization. EPCs phenotype was confirmed by immunofluorescence staining using uptake of Dil acetylated-LDL or binding of fluorescently labeled Ulex- europaeus agglutin 1 plant lectin.

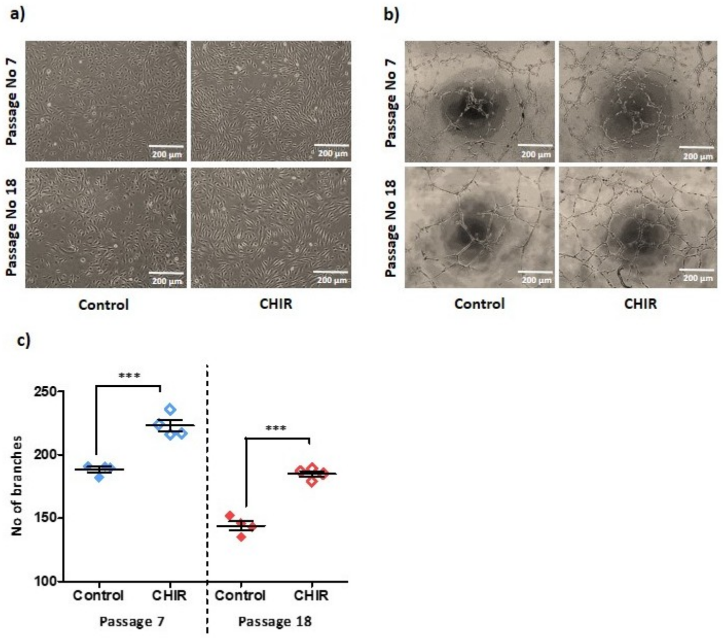

Figure S2. Treatment of CHIR99021 modulates late EPCs morphology and functional activities. (a) Late EPCs morphology during treatment with CHIR99021. (b) The tube forming ability was compared with young (p-7) and old (p-18) passage with and without CHIR99021in late EPCs. Data are presented as mean ± standard error of the mean (SEM). The results are considered statistically significant at * $p < 0.05$; ** $p < 0.01$; *** $p < 0.001$ when compared to untreated groups.

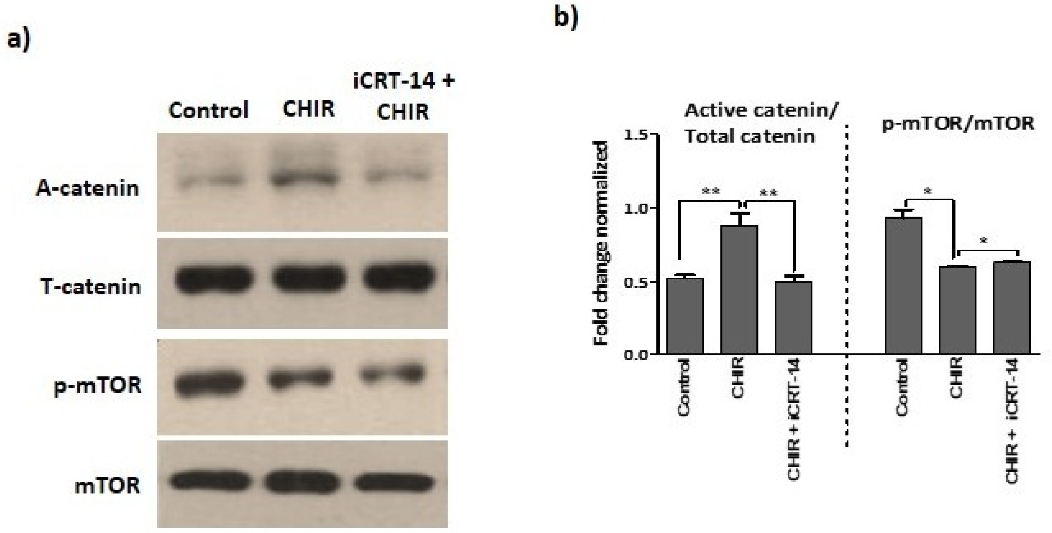

Figure S3. CHIR99021 induced mTOR inhibition is beta-catenin independent. Cells were treated with CHIR99021 (3 μM) alone, or co-treated with Beta-catenin inhibitor (10 μM) for 24 h. Then, western blot was performed to detect the protein level expression of active catenin, total catenin, p-mTOR, and mTOR (iCRT-14- beta-catenin inhibitor).
